# Supplementary material for: The Association between Vitamin D and Gut Microbiota: A Systematic Review of Human Studies
Source: Nutrients. 2021 Sep 26;13(10):3378. doi: 10.3390/nu13103378 (PMC8540279; doi:10.3390/nu13103378)
Supplement: Supplementary file 1 [file nutrients-13-03378-s001.zip › nutrients-1377761-supplementary.pdf]

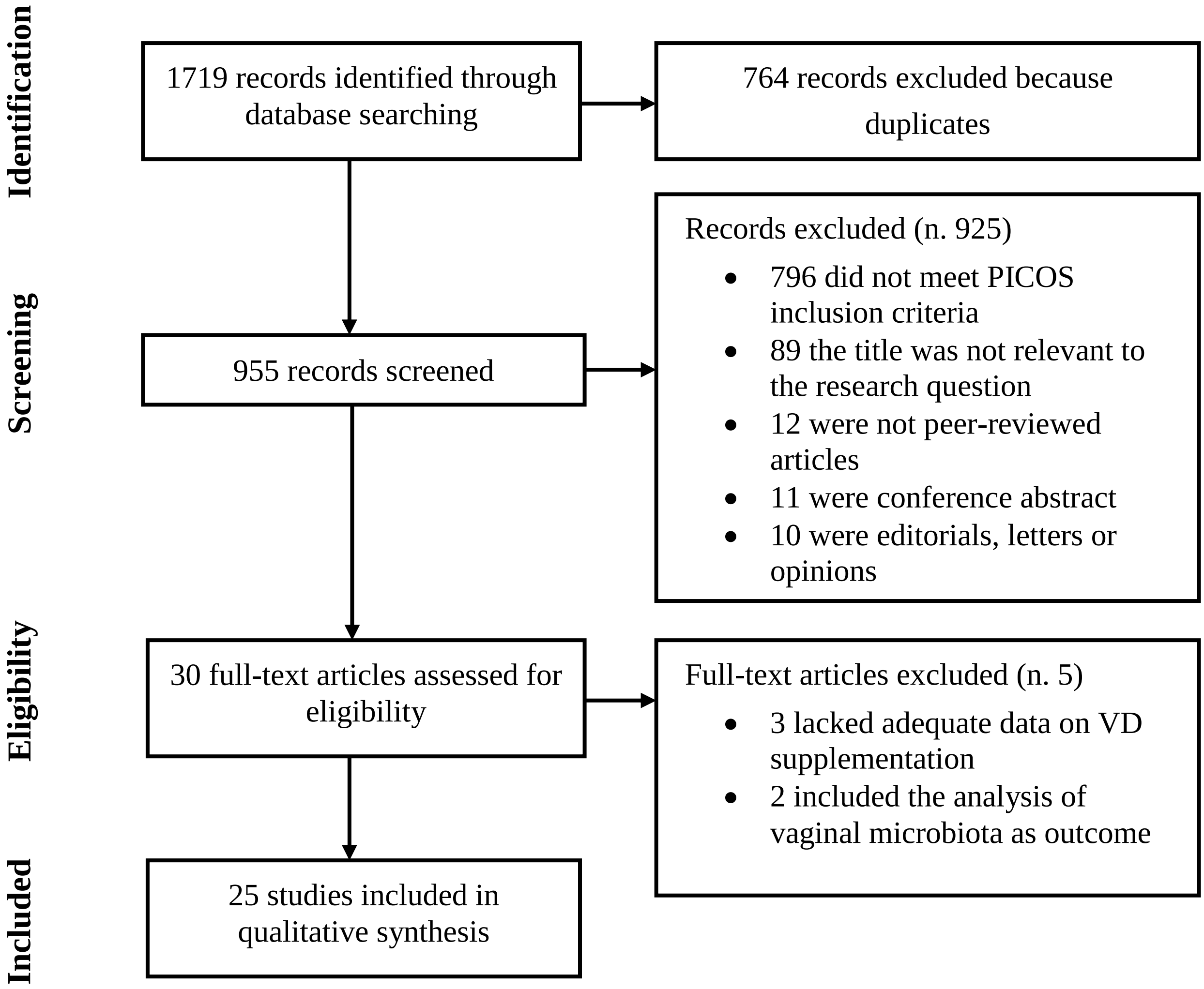

Figure S1: Flowchart of study selection

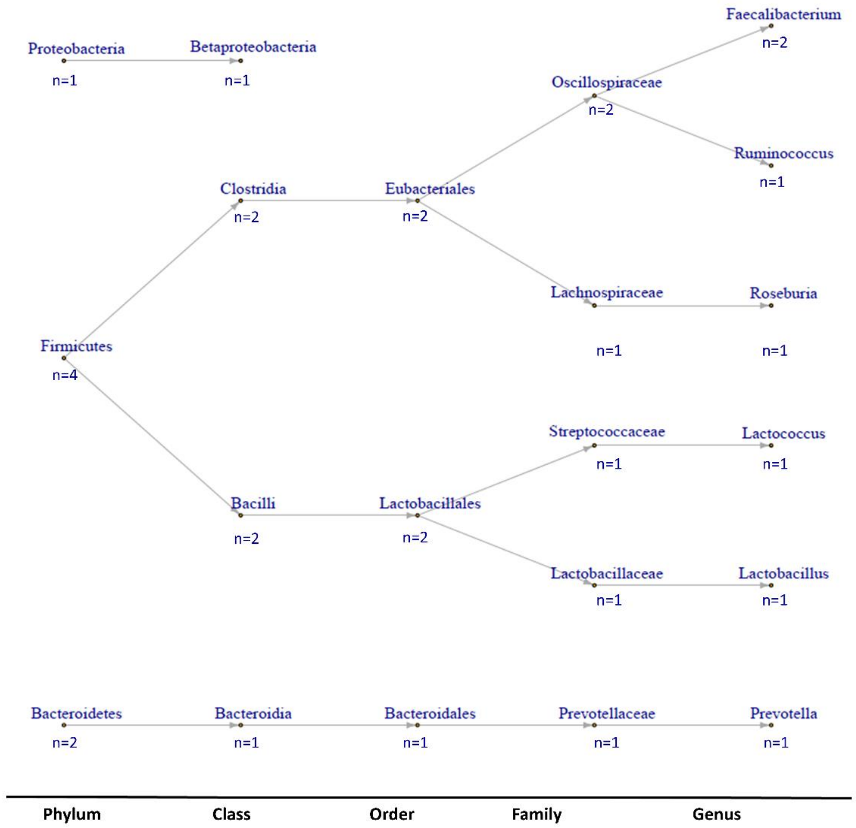

Figure S2. Phylogenetic tree of taxa that significantly decreased after vitamin D supplementation (supplementation group).

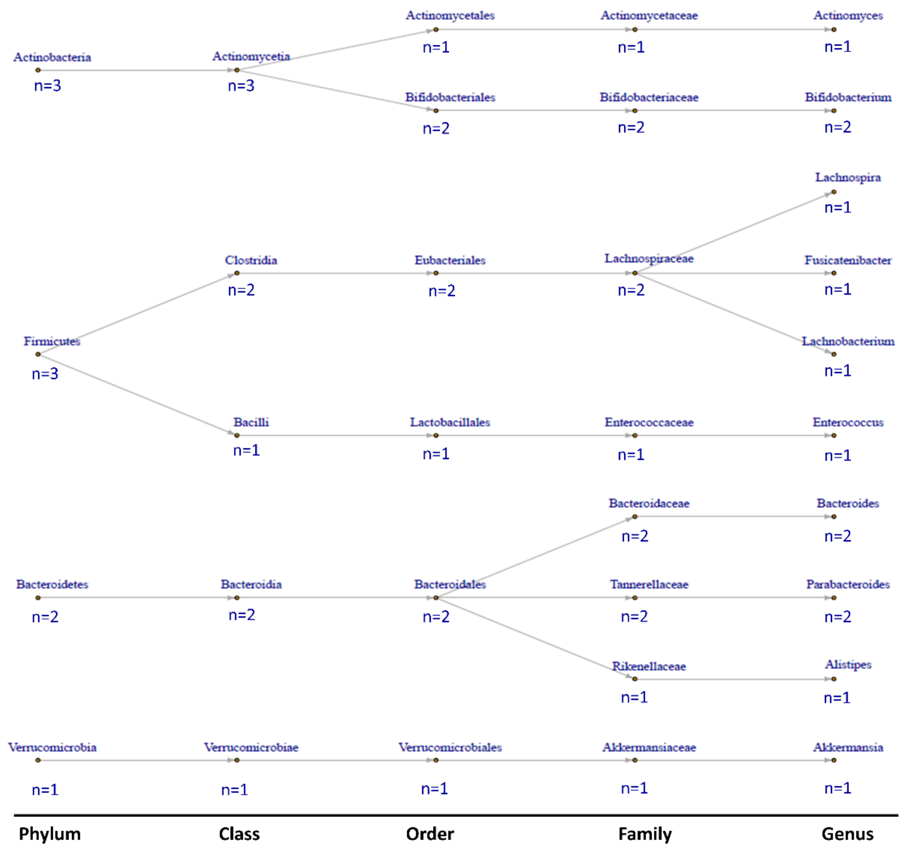

Figure S3. Phylogenetic tree of taxa that significantly increased after vitamin D supplementation (supplementation group).

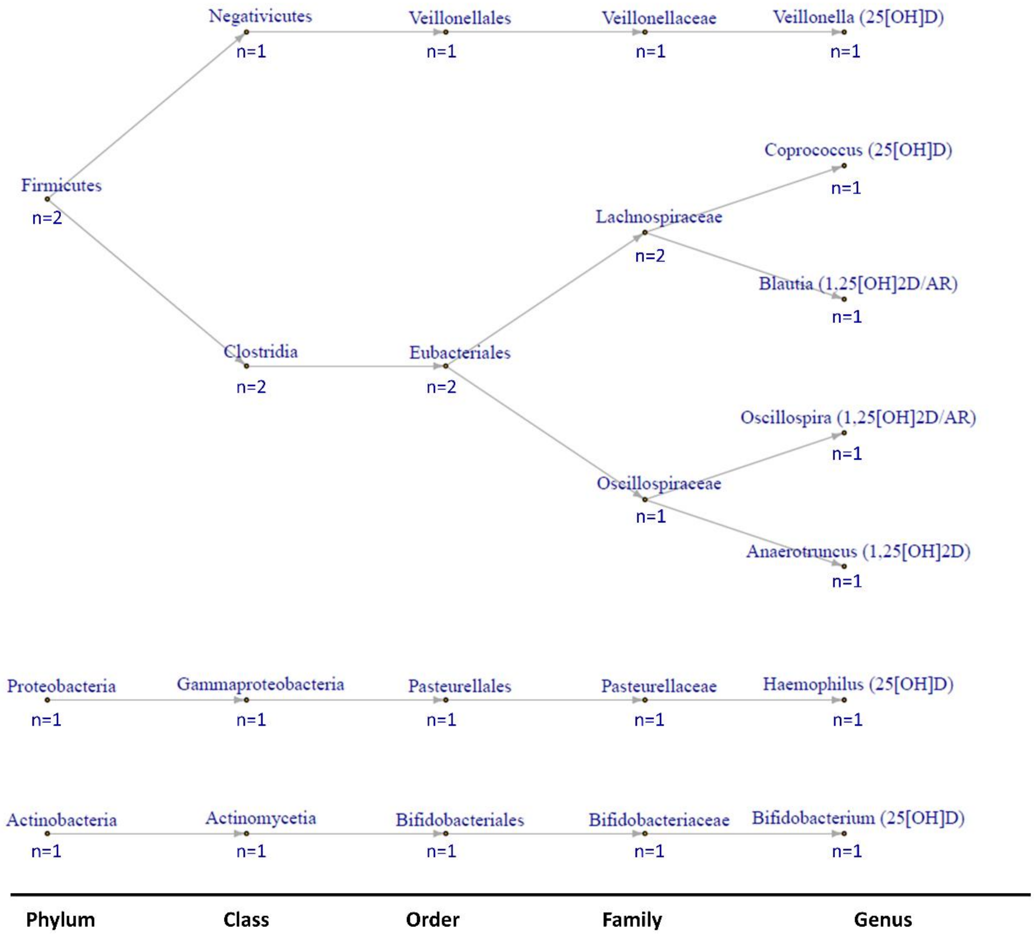

Figure S4. Phylogenetic tree of taxa that were significantly and negatively associated with either vitamin D serum concentrations or intake (non-supplementation group).

AR = Activation ratio of vitamin D, defined as 1,25(OH)2D/25(OH)D; 25(OH)D = 25 hydroxyvitamin D; 1,25(OH)2D = 1,25 hydroxyvitamin D2.

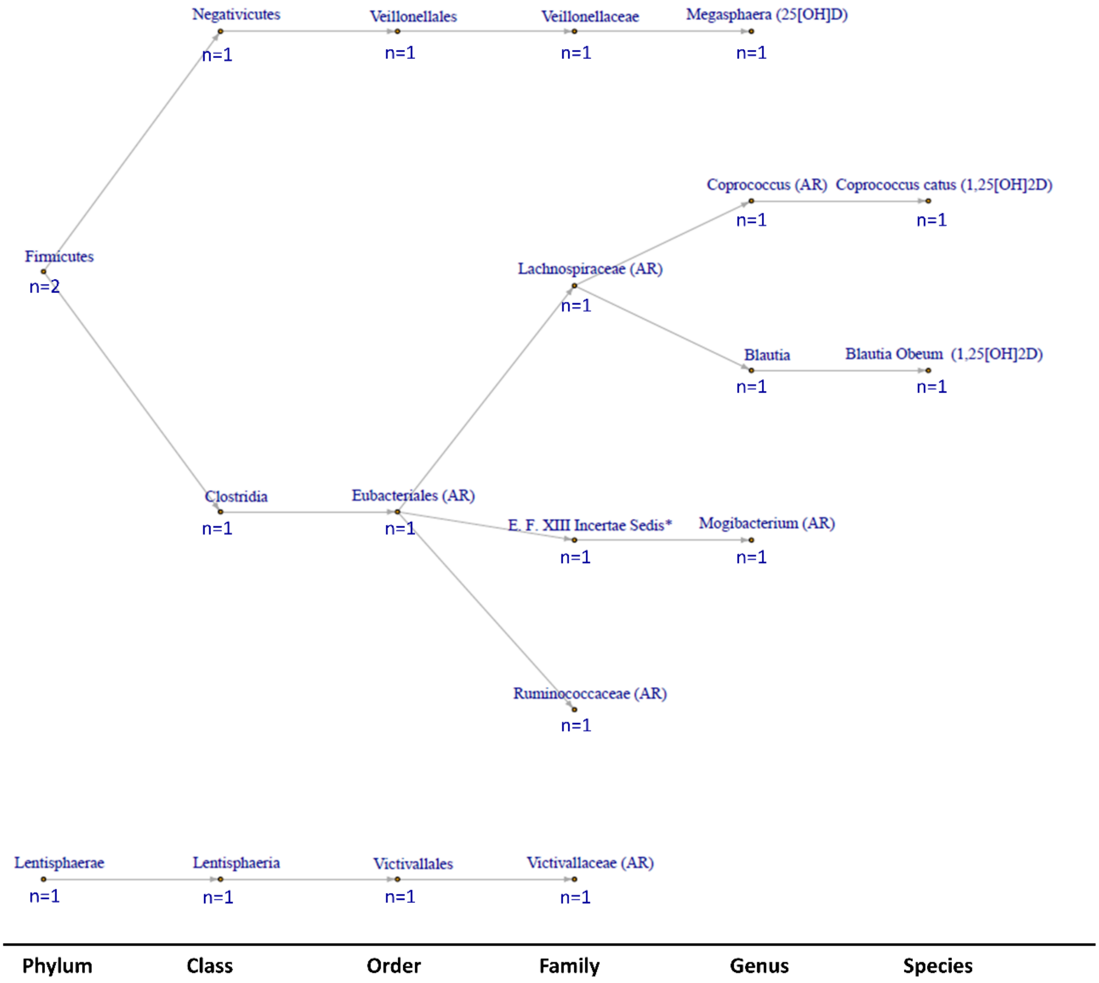

Figure S5. Phylogenetic tree of taxa that were significantly and positively associated with either vitamin D serum concentrations or intake (Non-supplementation group).

AR= Activation ratio of vitamin D, defined as 1,25(OH)2D/25(OH)D; 25(OH)D = 25 hydroxyvitamin D; 1,25(OH)2D = 1,25 hydroxyvitamin D2. *Eubacteriales Family XIII. Incertae Sedis.

Table S1. Phylogenetic reconstruction of taxa that significantly decreased after vitamin D supplementation (Supplementation group)

Author, PY	Health Status	Sample	Stratification	Phylum	Class	Order	Family	Genus	Species
Bashir, 2016	Healthy	Biopsy	Upper GI: GC (n paired = 13)	Proteobacteria	Gammaproteobacteria	Enterobacterales	Enterobacteriaceae	NA	Escherichia/Shigella
				Proteobacteria	Gammaproteobacteria	Pseudomonadales	Pseudomonadaceae	Pseudomonas	
				Firmicutes	Bacilli	Lactobacillales	Streptococcaceae	Lactococcus	
				Proteobacteria	Betaproteobacteria	Burkholderiales	Comamonadaceae	Variovorax	
				Proteobacteria	Gammaproteobacteria	Enterobacterales	Enterobacteriaceae	Enterobacteriaceae unclass	
				Proteobacteria	Gammaproteobacteria				
			Upper GI: GA (n paired = 13)	Proteobacteria	Gammaproteobacteria	Enterobacterales	Enterobacteriaceae	NA	Escherichia/Shigella
				Proteobacteria	Betaproteobacteria	Burkholderiales	Burkholderiaceae	Ralstonia	
				Proteobacteria	Gammaproteobacteria	Pseudomonadales	Pseudomonadaceae	Pseudomonas	
				Proteobacteria	Gammaproteobacteria	Xanthomonadales	Xanthomonadaceae	Stenotrophomonas	
				Proteobacteria	Gammaproteobacteria	Enterobacterales	Enterobacteriaceae	Enterobacteriaceae unclass	
				Proteobacteria	Gammaproteobacteria				
			Upper GI: DD (n paired = 13)	Proteobacteria	Gammaproteobacteria	Enterobacterales	Enterobacteriaceae	NA	Escherichia/Shigella
				Actinobacteria	Actinomycetia	Micrococcales	Microbacteriaceae	Leucobacter	
				Proteobacteria	Gammaproteobacteria	Pseudomonadales	Pseudomonadaceae	Pseudomonas	
			Lower GI: TI (n paired = 11)	Firmicutes	Clostridia	Eubacteriales	Peptostreptococcaceae	Peptostreptococcus	
			Lower GI: AO (n paired = 11)	Firmicutes	Clostridia			Clostridia unclass.	
			Lower GI: SC (n paired = 11)						
			Lower GI: AC (n paired = 12)						
		Stool	Stool (n paired = 8)	Proteobacteria	Betaproteobacteria				
Bosman,2019	Healthy (female)	Stool							
Cantarel, 2015	Healthy+MS (female)	Stool		Firmicutes	Clostridia	Eubacteriales	Oscillospiraceae	Ruminococcus	
	Healthy (female)								
	MS (female)		Untreated MS vs HC or treated MS	Proteobacteria	Gammaproteobacteria	Pseudomonadales	Moraxellaceae		
			Treated vs HC or treated MS	Firmicutes	Clostridia	Eubacteriales	Eubacteriaceae	Eubacterium	
				Firmicutes	Clostridia	Eubacteriales	Oscillospiraceae	Ruminococcus	
Charoenngam, 2020	Healthy	Stool		Firmicutes	Clostridia	Eubacteriales	Oscillospiraceae	Faecalibacterium	
				Firmicutes	Clostridia	Eubacteriales	Ruminococcaceae		
				Firmicutes	Clostridia				

PY = Publication Year; NA= Not Available; GI = gastrointestinal; GC = gastric corpus; GA = gastric antrum; DD = duodenum; TI = terminal ileum; AO = appendiceal orifice; AC = ascending colon; SC = sigmoid colon; MS = Multiple Sclerosis; HC = Healthy Controls; CD = Crohn disease; Q4 = upper quartile; Q1 = lower quartile.

Table S2. Phylogenetic reconstruction of taxa that significantly decreased after vitamin D supplementation (Supplementation group)

Author, PY	Health Status	Sample	Stratification	Phylum	Class	Order	Family	Genus	Species
Ciubotaru, 2015	Prediabetes (males)	Stool	25(OH)D (Q4 vs Q1)	Firmicutes	Clostridia	Eubacteriales	Oscillospiraceae	Ruminococcus	
				Firmicutes	Clostridia	Eubacteriales	Lachnospiraceae	Blautia	
				Firmicutes	Clostridia	Eubacteriales	Lachnospiraceae	Roseburia	
				Firmicutes	Clostridia	Eubacteriales	Lachnospiraceae		
			Delta 25(OH)D (Q4 vs Q1)	Firmicutes	Clostridia	Eubacteriales	Oscillospiraceae	Ruminococcus	
				Firmicutes	Clostridia	Eubacteriales	Lachnospiraceae	Blautia	
				Firmicutes	Clostridia	Eubacteriales	Lachnospiraceae	Roseburia	
				Firmicutes	Clostridia	Eubacteriales	Lachnospiraceae	Dorea	
				Firmicutes	Clostridia	Eubacteriales	Lachnospiraceae		
Drall, 2020	Pregnancy (infants)	Stool	Infant vit D supplementation	Firmicutes	Negativicutes	Selenomonadales	Selenomonadaceae	Megamonas	
				Firmicutes	Negativicutes	Veillonellales	Veillonellaceae		
			Maternal prenatal or postnatal vit D suppl	Proteobacteria	Deltaproteobacteria	Desulfovibrionales	Desulfovibrionaceae	Bilophila (only breastfed)	
				Firmicutes	Clostridia	Eubacteriales	Lachnospiraceae	Other (only brestfed)	
Garg, 2018		Stool							
Hjelmsø, 2020	Pregnancy	Infant stool							
Kanhere, 2018	Cystic fibrosis	Stool	Stool: vit D sufficient vs vit D insufficient at baseline	Proteobacteria	Gammaproteobacteria				
		Stool	Stool: change in microbiota after supplementation	Firmicutes	Clostridia	Eubacteriales	Oscillospiraceae	Anaerotruncus	
				Firmicutes	Negativicutes	Veillonellales	Veillonellaceae	Veillonella	
				Firmicutes	Clostridia	Eubacteriales	Clostridiaceae		
				Firmicutes	Erysipelotrichia	Erysipelotrichales	Erysipelotrichaceae		
Missailidis,2019	HIV	Biopsy							
Naderpoor, 2018	Obesity	Stool	Vit D suppl. vs Placebo at follow-up	Firmicutes	Clostridia	Eubacteriales	Lachnospiraceae	Blautia	
			25(OH)D>75 nmol/L vs 25(OH)D<50 nmol/L at follow-up	Firmicutes	Clostridia	Eubacteriales	Oscillospiraceae	Ruminococcus	
				Firmicutes	Clostridia	Eubacteriales	Clostridiaceae		
Schaffler, 2018	Crohn disease; Healthy	Stool	CD: Week 4						
			HC						
Singh,2020	Healthy (female)	Stool	Main analysis	Firmicutes	Clostridia	Eubacteriales	Lachnospiraceae	Roseburia	
				Firmicutes	Clostridia	Eubacteriales	Oscillospiraceae	Ruminococcus	
				Firmicutes	Clostridia	Eubacteriales	Oscillospiraceae	Faecalibacterium	
				Bacteroidetes	Bacteroidia	Bacteroidales	Prevotellaceae	Prevotella	
				Firmicutes					

PY = Publication Year; NA= Not Available; 25(OH)D = 25 hydroxyvitamin D; HC = Healthy Controls; CD = Crohn Disease; Q4 = upper quartile; Q1 = lower quartile.

Table S3. Phylogenetic reconstruction of taxa that significantly decreased after vitamin D supplementation (Supplementation group)

Author, PY	Health Status	Sample	Stratification	Phylum	Class	Order	Family	Genus	Species
Singh,2020	Healthy (female)	Stool	Responders (>20 ng/ml) vs non-responders (<20 ng/ml)						
			Responders	Firmicutes					
			Non-responders	Proteobacteria					
Sordillo,2016	Healthy	Stool		Firmicutes	Bacilli	Lactobacillales	Streptococcaceae	Lactococcus	
Tabatabaeizadeh, 2019	Healthy (female, adolescents)	Stool		Firmicutes	Bacilli	Lactobacillales	Lactobacillaceae	Lactobacillus	
				Bacteroidetes					
Talsness, 2017	Pregnancy (infants)	Stool	Vit D supplementation (none, <10mg, >=10mg)	Actinobacteria	Actinomycetia	Bifidobacteriales	Bifidobacteriaceae	Bifidobacterium	Bifidobacterium sp
			25(OH) levels (quintiles)	Actinobacteria	Actinomycetia	Bifidobacteriales	Bifidobacteriaceae	Bifidobacterium	Bifidobacterium sp
				Bacteroidetes	Bacteroidia	Bacteroidales	Bacteroidaceae	Bacteroides	Bacteroides fragilis
			Infant vit D suppl. (yes vs no)						

PY = Publication Year; NA= Not Available; 25(OH)D = 25 hydroxyvitamin D; vit D = vitamin D.

Table S4. Phylogenetic reconstruction of taxa that significantly increased after vitamin D supplementation (Supplementation group)

Author, PY	Health Status	Sample	Stratification	Phylum	Class	Order	Family	Genus	Species
Bashir, 2016	Healthy	Biopsy	Upper GI: GC (n paired = 13)	Proteobacteria	Alphaproteobacteria	Hyphomicrobiales	Bradyrhizobiaceae	Bradyrhizobium	
				Proteobacteria	Epsilonproteobacteria	Campylobacterales	Campylobacteraceae	Sulfurospirillum	
				Actinobacteria	Actinomycetia	Actinomycetales	Actinomycetaceae	Actinomyces	
			Upper GI: GA (n paired = 13)	Firmicutes	Bacilli	Lactobacillales	Carnobacteriaceae	Alkalibacterium	
				Proteobacteria	Alphaproteobacteria	Hyphomicrobiales	Bradyrhizobiaceae	Bradyrhizobium	
				Proteobacteria	Alphaproteobacteria				
			Upper GI: DD (n paired = 13)	Proteobacteria	Alphaproteobacteria	Hyphomicrobiales	Bradyrhizobiaceae	Bradyrhizobium	
				Proteobacteria	Betaproteobacteria	Burkholderiales	Oxalobacteraceae	Janthinobacterium	
				Proteobacteria	Gammaproteobacteria	Oceanospirillales	Halomonadaceae	Halomonas	
				Bacteroidetes				Bacteroidetes unclass.	
			Lower GI: TI (n paired = 11)	Firmicutes	Clostridia	Eubacteriales	Lachnospiraceae	Roseburia	
			Lower GI: AO (n paired = 11)						
			Lower GI: SC (n paired = 11)						
			Lower GI: AC (n paired = 12)						
		Stool	Stool (n paired = 8)	Actinobacteria	Actinomycetia	Actinomycetales	Actinomycetaceae	Actinomyces	
Bosman,2019	Healthy (female)	Stool		Firmicutes	Clostridia	Eubacteriales	Lachnospiraceae	Lachnospira	
				Firmicutes	Clostridia	Eubacteriales	Lachnospiraceae	Fusicatenibacter	
				Firmicutes	Clostridia	Eubacteriales	Lachnospiraceae		
Cantarel, 2015	Healthy+MS (female)	Stool		Firmicutes	Clostridia	Eubacteriales	Oscillospiraceae	Faecalibacterium	
				Proteobacteria	Gammaproteobacteria	Enterobacterales	Enterobacteriaceae		
	Healthy (female)								
	Multiple Sclerosys (female)		Untreated MS vs HC or treated MS	Verrucomicrobia	Verrucomicrobiae	Verrucomicrobiales	Akkermansiaceae	Akkermansia	
				Firmicutes	Clostridia	Eubacteriales	Oscillospiraceae	Faecalibacterium	
				Firmicutes	Clostridia	Eubacteriales	Lachnospiraceae	Coprococcus	
			Treated vs HC or treated MS	Proteobacteria	Betaproteobacteria	Burkholderiales	Oxalobacteraceae	Janthinobacterium	
Charoenngam, 2020	Healthy	Stool		Bacteroidetes	Bacteroidia	Bacteroidales	Bacteroidaceae	Bacteroides	
				Bacteroidetes	Bacteroidia	Bacteroidales	Tannerellaceae	Parabacteroides	
Ciubotaru, 2015	Prediabetes (males)	Stool	25(OH)D (Q4 vs Q1)						
			Delta 25(OH)D (Q4 vs Q1)						
Drall, 2020	Pregnancy (infants)	Stool	Infant vit D suppl.						
			Maternal prenatal or postnatal vit D suppl	Proteobacteria	Gammaproteobacteria	Pasteurellales	Pasteurellaceae	Haemophilus (only breastfed)	

PY = Publication Year; NA= Not Available; GI = gastrointestinal; GC = gastric corpus; GA = gastric antrum; DD = duodenum; TI = terminal ileum; AO = appendiceal orifice; AC = ascending colon; SC = sigmoid colon; MS = Multiple Sclerosis; HC = Healthy Controls; CD = Crohn disease; Q4 = upper quartile; Q1 = lower quartile.

Table S5. Phylogenetic reconstruction of taxa that significantly increased after vitamin D supplementation (Supplementation group)

Author, PY	Health Status	Sample	Stratification	Phylum	Class	Order	Family	Genus	Species
Garg, 2018		Stool		Firmicutes	Clostridia	Eubacteriales	Clostridiaceae	Clostridium	Clostridium colinae
				Proteobacteria	Gammaproteobacteria	Enterobacterales	Enterobacteriaceae		
Hjelmsø, 2020	Pregnancy	Infant stool							
Kanhere, 2018	Cystic fibrosis	Stool	Stool: vit D sufficient vs vit D insufficient at baseline	Bacteroidetes	Bacteroidia	Bacteroidales	Bacteroidaceae	Bacteroides	
				Bacteroidetes	Bacteroidia	Bacteroidales	Tannerellaceae	Parabacteroides	
				Bacteroidetes	Bacteroidia	Bacteroidales	Bacteroidaceae		
				Bacteroidetes	Bacteroidia	Bacteroidales	Porphyromonadaceae		
		Stool	Stool: change in microbiota after suppl.	Firmicutes	Bacilli	Lactobacillales	Streptococcaceae	Lactococcus	
				Firmicutes	Clostridia	Eubacteriales	Oscillospiraceae	Ruminococcus	
				Firmicutes	Negativicutes	Acidaminococcales	Acidaminococcaceae	Acidaminococcus	
				Firmicutes	Negativicutes	Acidaminococcales	Acidaminococcaceae	Phascolarctobacterium	
				Bacteroidetes	Bacteroidia	Bacteroidales	Odoribacteraceae		
				Bacteroidetes	Bacteroidia	Bacteroidales	Paraprevotellaceae		
Missailidis,2019	HIV	Biopsy							
Naderpoor, 2018	Obesity	Stool	Vit D suppl. vs Placebo at follow-up	Firmicutes	Clostridia	Eubacteriales	Lachnospiraceae	Lachnospira	
			25(OH)D>75 nmol/L vs 25(OH)D<50 nmol/L at fullow-up	Firmicutes	Clostridia	Eubacteriales	Lachnospiraceae	Coprococcus	Coprococcus eutactus
				Firmicutes	Clostridia	Eubacteriales	Lachnospiraceae	Coprococcus	
Schaffler, 2018	Crohn disease; Healthy	Stool	CD: Week 4	Firmicutes	Bacilli	Lactobacillales	Lactobacillaceae	Lactobacillus	
				Firmicutes	Negativicutes	Veillonellales	Veillonellaceae	Megasphaera	
			HC						
Singh,2020	Healthy (female)	Stool	Main analysis	Actinobacteria	Actinomycetia	Bifidobacteriales	Bifidobacteriaceae	Bifidobacterium	
				Verrucomicrobia	Verrucomicrobiae	Verrucomicrobiales	Akkermansiaceae	Akkermansia	
				Bacteroidetes	Bacteroidia	Bacteroidales	Bacteroidaceae	Bacteroides	
				Bacteroidetes	Bacteroidia	Bacteroidales	Rikenellaceae	Alistipes	
				Bacteroidetes	Bacteroidia	Bacteroidales	Tannerellaceae	Parabacteroides	
				Bacteroidetes					
			Responders (>20 ng/ml) vs non-responders (<20 ng/ml)	Bacteroidetes	Bacteroidia	Bacteroidales	Bacteroidaceae	Bacteroides	Bacteroides acidifaciens
				Firmicutes	Clostridia	Eubacteriales	Oscillospiraceae	Ruminococcus	Ruminococcus bromii
				Bacteroidetes	Bacteroidia	Bacteroidales	Bacteroidaceae	Bacteroides	Bacteroides eggerthii
				Bacteroidetes	Bacteroidia	Bacteroidales	Barnesiellaceae	Barnesiella	Barnesiella intestinihominis

PY = Publication Year; 25(OH)D = 25 hydroxyvitamin D; HC = Healthy Controls; CD = Crohn Disease; vit D = vitamin D.

Table S6. Phylogenetic reconstruction of taxa that significantly increased after vitamin D supplementation (Supplementation group)

Author, PY	Health Status	Sample	Stratification	Phylum	Class	Order	Family	Genus	Species
Singh,2020	Healthy (female)	Stool	Responders	Bacteroidetes					
				Actinobacteria					
				Proteobacteria					
				Lentisphaeraea					
			Non-responders	Firmicutes	Clostridia	Eubacteriales	Lachnospiraceae	Roseburia	Roseburia faecis
				Bacteroidetes	Bacteroidia	Bacteroidales	Bacteroidaceae	Bacteroides	Bacteroides eggerthii
				Bacteroidetes	Bacteroidia	Bacteroidales	Prevotellaceae	Prevotella	Prevotella copri
				Firmicutes	Clostridia	Eubacteriales	Oscillospiraceae	Oscillospira	Oscillospira guilliermondii
				Bacteroidetes	Bacteroidia	Bacteroidales	Rikenellaceae	Alistipes	Alistipes finegoldii
Sordillo,2016	Healthy	Stool		Firmicutes	Clostridia	Eubacteriales	Lachnospiraceae	Lachnobacterium	
Tabatabaeizadeh, 2019	Healthy (female, adolescents)	Stool		Firmicutes	Bacilli	Lactobacillales	Enterococcaceae	Enterococcus	
				Actinobacteria	Actinomycetia	Bifidobacteriales	Bifidobacteriaceae	Bifidobacterium	
				Firmicutes					
Talsness, 2017	Pregnancy (infants)	Stool	Vit D supplementation (none, <10mg, >=10mg)						
			25(OH) levels (quintiles)						
			Infant vit D suppl. (yes vs no)						

PY = Publication Year; 25(OH)D = 25 hydroxyvitamin D; vit D = vitamin D.

Table S7. Phylogenetic reconstruction of taxa that were significantly and negatively associated with either vitamin D serum concentrations or intake (Non-supplementation group)

Author, PY	Health Status	Vit D	Sample	Stratification	Phylum	Class	Order	Family	Genus	Species
Kassem, 2020	Pregnancy	Prenatal maternal 25[OH]D and cord 25[OH]D	Stool	Prenatal maternal 25(OH)D	Firmicutes	Tissierellia	Tissierellales	Peptoniphilaceae	Anaerococcus	
					Actinobacteria	Actinomycetia	Bifidobacteriales	Bifidobacteriaceae	Bifidobacterium	
				Cord 25(OH)D	Firmicutes	Clostridia	Eubacteriales	Lachnospiraceae	Mediterraneibacter	Ruminococcus gnavus
Luthold, 2017	Healthy	Dietary vit D intake	Stool	Dietary Vit D intake tertiles	Firmicutes	Negativicutes	Veillonellales	Veillonellaceae	Veillonella	
					Proteobacteria	Gammaproteobacteria	Pasteurellales	Pasteurellaceae	Haemophilus	
Luthold, 2017	Healthy	25(OH)D	Stool	25(OH)D concentrations tertiles	Firmicutes	Negativicutes	Veillonellales	Veillonellaceae	Veillonella	
					Proteobacteria	Gammaproteobacteria	Pasteurellales	Pasteurellaceae	Haemophilus	
					Firmicutes	Clostridia	Eubacteriales	Lachnospiraceae	Coprococcus	
					Actinobacteria	Actinomycetia	Bifidobacteriales	Bifidobacteriaceae	Bifidobacterium	
Mandal, 2016	Pregnancy	Dietary vit D intake	Stool	Maternal microbiota	Bacteroidetes					
Seura, 2017	Healthy (female)	Dietary vit D intake	Stool							
Soltys, 2020	Ulcerative Colitis	Serum Vit D levels	Stool	Stool						
			Biopsy	Biopsy: sigma inflamed	Proteobacteria	Gammaproteobacteria	Pasteurellales	Pasteurellaceae	Haemophilus	Haemophilus parainfluenzae
					Firmicutes	Bacilli	Lactobacillales	Streptococcaceae	Streptococcus	
					Fusobacteria	Fusobacteriia	Fusobacteriales	Fusobacteriaceae	Fusobacterium	
					Firmicutes	Bacilli	Lactobacillales	Streptococcaceae		
					Proteobacteria	Gammaproteobacteria	Pasteurellales	Pasteurellaceae		
					Fusobacteria	Fusobacteriia	Fusobacteriales	Fusobacteriaceae		
					Proteobacteria	Gammaproteobacteria	Pasteurellales			
					Fusobacteria	Fusobacteriia	Fusobacteriales			
					Fusobacteria					
				Biopsy: sigma non-inflamed	Actinobacteria	Coriobacteriia	Coriobacteriales	Coriobacteriaceae	Collinsella	Collinsella aerofaciens
					Fusobacteria	Fusobacteriia	Fusobacteriales	Fusobacteriaceae	Fusobacterium	
					Fusobacteria	Fusobacteriia	Fusobacteriales	Fusobacteriaceae		
					Fusobacteria	Fusobacteriia	Fusobacteriales			
					Actinobacteria					
					Fusobacteria					

PY = Publication Year; 25(OH)D = 25 hydroxyvitamin D; vit D = vitamin D.

Table S8. Phylogenetic reconstruction of taxa that were significantly and negatively associated with either vitamin D serum concentrations or intake (Non-supplementation group)

Author, PY	Health Status	Vit D	Sample	Stratification	Phylum	Class	Order	Family	Genus	Species
Soltys, 2020	Crohn disease	25(OH)D	Stool	Stool						
			Biopsy	Biopsy: sigma inflamed	Firmicutes					
				Biopsy: sigma non-inflamed						
				Biopsy: terminal ileum inflamed						
				Biopsy: terminal ileum non-inflamed						
Thomas, 2020	Healthy (male, older)	25(OH)D; 1,25(OH)$_2$D; 24,25(OH)$_2$D; activation ratio (1,25(OH)$_2$D/25(OH)D) and catabolism ratio	Stool	1,25(OH)$_2$D	Firmicutes	Clostridia	Eubacteriales	Oscillospiraceae	Oscillospira	
					Firmicutes	Clostridia	Eubacteriales	Lachnospiraceae	Blautia	
					Firmicutes	Clostridia	Eubacteriales	Oscillospiraceae	Anaerotruncus	
				Activation ratio	Firmicutes	Clostridia	Eubacteriales	Oscillospiraceae	Oscillospira	
					Firmicutes	Clostridia	Eubacteriales	Lachnospiraceae	Blautia	
Weng, 2019	Ulcerative Colitis; Healthy controls	Dietary vit D intake	Biopsy		Firmicutes	Clostridia	Eubacteriales	Lachnospiraceae	Dorea	
					Firmicutes	Clostridia	Eubacteriales	Oscillospiraceae	Ruminococcus 2	
			Stool		Firmicutes	Clostridia	Eubacteriales	Clostridiaceae	Clostridium	Clostridium clostridioforme CAG:132
	Crohn disease; Healthy controls	Dietary vit D intake	Biopsy		Firmicutes	Bacilli	Lactobacillales	Lactobacillaceae	Lactobacillus	
					Actinobacteria	Actinomycetia	Micrococcales	Intrasporangiaceae	Janibacter	
					Proteobacteria	Hydrogenophilalia	Hydrogenophilales	Hydrogenophilaceae	Hydrogenophilus	
			Stool							
Wu, 2011	Healthy	Dietary Vit D intakes	Stool		Firmicutes	Negativicutes	Veillonellales	Veillonellaceae	Dialister	

PY = Publication Year; 25(OH)D = 25 hydroxyvitamin D; 1,25(OH)$_2$D = 1,25 hydroxyvitamin D2; 24,25(OH)$_2$D = 24,25 hydroxyvitamin D_2; vit D = vitamin D.

Table S9. Phylogenetic reconstruction of taxa that were significantly and positively associated with either vitamin D serum concentrations or intake (Non-supplementation group)

Author, PY	Health Status	Vit D	Sample	Stratification	Phylum	Class	Order	Family	Genus	Species
Kassem, 2020	Pregnancy	Prenatal maternal 25(OH)D and cord 25(OH)D	Stool	Prenatal maternal 25(OH)D	Firmicutes	Clostridia	Eubacteriales	Lachnospiraceae	Mediterraneibacter	Ruminococcus gnavus
					Proteobacteria	Gammaproteobacteria	Pseudomonadales	Moraxellaceae	Acinetobacter	
					Actinobacteria	Actinomycetia	Corynebacteriales	Corynebacteriaceae	Corynebacterium	
					Firmicutes	Clostridia	Eubacteriales	Clostridiaceae		
				Cord 25(OH)D	Proteobacteria	Gammaproteobacteria	Pseudomonadales	Moraxellaceae	Acinetobacter	Acinetobacter rhizosphaerae
					Proteobacteria	Gammaproteobacteria	Pseudomonadales	Moraxellaceae	Acinetobacter	
					Firmicutes	Erysipelotrichia	Erysipelotrichales	Erysipelotrichaceae	Bulleidia	
					Actinobacteria	Actinomycetia	Corynebacteriales	Corynebacteriaceae	Corynebacterium	
					Firmicutes	Tissierellia	Tissierellales	Peptoniphilaceae	Finegoldia	
					Firmicutes	Tissierellia	Tissierellales	Peptoniphilaceae	Peptoniphilus	
					Firmicutes	Bacilli	Lactobacillales	Streptococcaceae	Streptococcus	
					Firmicutes	Clostridia	Eubacteriales	Clostridiaceae		
					Proteobacteria	Gammaproteobacteria	Enterobacterales	Enterobacteriaceae		
Luthold, 2017	Healthy	Dietary vit D intake	Stool	Dietary Vit D intake tertiles	Bacteroidetes	Bacteroidia	Bacteroidales	Prevotellaceae	Prevotella	
Luthold, 2017	Healthy	25(OH)D	Stool	25(OH)D concentrations tertiles	Firmicutes	Negativicutes	Veillonellales	Veillonellaceae	Megasphaera	
Mandal, 2016	Pregnancy	Dietary vit D intake	Stool	Maternal microbiota	Firmicutes	Bacilli	Bacillales	Staphylococcaceae	Staphylococcus	
Seura, 2017	Healthy (female)	Dietary vit D intake	Stool/Biopsy							
Soltys, 2020	Ulcerative Colitis	25(OH)D	Stool	Stool						
			Biopsy	Biopsy: sigma inflamed						
				Biopsy: sigma non-inflamed						
Soltys, 2020	Crohn disease	25(OH)D	Stool	Stool	Proteobacteria	Gammaproteobacteria	Pasteurellales	Pasteurellaceae	Haemophilus	
					Proteobacteria	Gammaproteobacteria	Pasteurellales	Pasteurellaceae		
					Proteobacteria	Gammaproteobacteria	Pasteurellales			
			Biopsy	Biopsy: sigma inflamed						
				Biopsy: sigma non-inflamed						
				Biopsy: terminal ileum inflamed						
				Biopsy: terminal ileum non-inflamed						

PY = Publication Year; 25(OH)D = 25 hydroxyvitamin D; vit D = vitamin D intake.

Table S10. Phylogenetic reconstruction of taxa that were significantly and positively associated with either vitamin D serum concentrations or intake (Non-supplementation group)

Author, PY	Health Status	Vit D	Sample	Stratification	Phylum	Class	Order	Family	Genus	Species
Thomas, 2020	Healthy (male, older)	25(OH)D; 1,25(OH)2D; 24,25(OH)2D; activation ratio (1,25(OH)2D/25(OH)D) and catabolism ratio	Stool	1,25(OH)2D	Firmicutes	Clostridia	Eubacteriales	Lachnospiraceae	Coprococcus	Coprococcus catus
					Firmicutes	Clostridia	Eubacteriales	Lachnospiraceae	Blautia	Blautia Obeum
				Activation ratio	Firmicutes	Clostridia	Eubacteriales	Eubacteriales Family XIII. Incertae Sedis	Mogibacterium	
					Firmicutes	Clostridia	Eubacteriales	Lachnospiraceae	Coprococcus	
					Firmicutes	Clostridia	Eubacteriales	Ruminococcaceae		
					Firmicutes	Clostridia	Eubacteriales	Lachnospiraceae		
					Lentisphaerae	Lentisphaeria	Victivallales	Victivallaceae		
					Firmicutes	Clostridia	Eubacteriales			
Weng, 2019	Ulcerative Colitis; Healthy controls	Dietary vit D intake	Biopsy		Proteobacteria	Deltaproteobacteria	Desulfovibrionales	Desulfovibrionaceae	Bilophila	
			Stool		Proteobacteria	Deltaproteobacteria	Desulfovibrionales	Desulfovibrionaceae	Desulfovibrio	
					Bacteroidetes	Bacteroidia	Bacteroidales	Barnesiellaceae	Barnesiella	
	Crohn disease; Healthy controls	Dietary vit D intake	Biopsy		Firmicutes	Clostridia	Eubacteriales	Lachnospiraceae	Fusicatenibacter	
					Firmicutes	Clostridia	Eubacteriales	Lachnospiraceae	Blautia	
					Firmicutes	Clostridia	Eubacteriales	Lachnospiraceae	Lachnospiracea incertae sedis	
			Stool		Firmicutes	Clostridia	Eubacteriales	Oscillospiraceae	Ruminococcus	
					Firmicutes	Clostridia	Eubacteriales	Lachnospiraceae	Fusicatenibacter	
					Proteobacteria	Oligoflexia	Bdellovibrionales	Bdellovibrionaceae	Bdellovibrio	
					Bacteroidetes	Bacteroidia	Bacteroidales	Barnesiellaceae	Barnesiella	
Wu, 2011	Healthy	Dietary Vit D intakes	Stool		Bacteroidetes	Bacteroidia	Bacteroidales	Bacteroidaceae	Bacteroides	

PY = Publication Year; 25(OH)D = 25 hydroxyvitamin D; 1,25$(OH)_2$D = 1,25 hydroxyvitamin D2; 24,25$(OH)_2$D = 24,25 hydroxyvitamin D_2; vit D = vitamin D.
